# ASO Author Reflections: Percutaneous Hepatic Perfusion with Melphalan in Patients with Unresectable Hepatic Metastases from Ocular Melanoma

**DOI:** 10.1245/s10434-020-08806-x

**Published:** 2020-07-18

**Authors:** T. Susanna Meijer, Mark C. Burgmans

**Affiliations:** Department of Radiology, Leiden University Medical Center, Leiden, The Netherlands

## Past

Up to 50% of patients with ocular melanoma will develop metastatic disease with predominant liver involvement. Because effective systemic treatments are lacking, liver-directed therapies play a key role in the management of these patients. The superiority of percutaneous hepatic perfusion with melphalan (M-PHP) over best available care in controlling liver disease has been demonstrated in a randomized, controlled trial.^1^ In this study, approximately 40% of patients had extrahepatic disease and M-PHP was associated with high rates of hematologic toxicity. To reduce the rate of hematologic toxicity following M-PHP, a new hemofiltration system with a second-generation detoxification cartridge (GEN 2 filter) was developed. So far, only retrospective studies have been published on this subject.^2^–^4^

## Present

This prospective, phase II study investigated the efficacy and safety of M-PHP using the GEN 2 filter in well-selected patients with unresectable metastases from ocular melanoma confined to the liver.^5^ Sixty-four M-PHP procedures were performed in 35 patients. The overall response rate of 72% (complete response 3%, partial response 69%) and median overall survival (OS) of 19.1 months in the current study appeared to be more favourable compared with published data on other treatment modalities and provide convincing evidence for the efficacy of M-PHP in metastatic ocular melanoma. Furthermore, responders demonstrated a significantly longer median OS than nonresponders (27.5 vs. 11.9 months, *p *< 0.001). M-PHP is a well-tolerated procedure with an acceptable safety profile.

## Future

Seventy-four percent of patients in this study developed extrahepatic metastatic disease during follow-up. This indicates that many patients with ocular melanoma will suffer from systemic spread for which liver-directed therapy is only a temporarily solution. We recently started a phase I/II study investigating combination therapy of M-PHP + ipilimumab/nivolumab to better control both hepatic and extrahepatic disease (CHOPIN trial, NCT04283890). Future research should try to reproduce abovementioned results in a large, multicenter trial and to develop standardized patient selection criteria.
